# Population pharmacokinetics of ethambutol in African children: a pooled analysis

**DOI:** 10.1093/jac/dkac127

**Published:** 2022-04-25

**Authors:** Tjokosela Tikiso, Helen McIlleron, Mahmoud Tareq Abdelwahab, Adrie Bekker, Anneke Hesseling, Chishala Chabala, Geraint Davies, Heather J Zar, Helena Rabie, Isabelle Andrieux-Meyer, Janice Lee, Lubbe Wiesner, Mark F Cotton, Paolo Denti

**Affiliations:** Division of Clinical Pharmacology, Department of Medicine, University of Cape Town, Cape Town, South Africa; Division of Clinical Pharmacology, Department of Medicine, University of Cape Town, Cape Town, South Africa; Wellcome Centre for Infectious Diseases Research in Africa (CIDRI-Africa), Institute of Infectious Disease and Molecular Medicine, University of Cape Town, Cape Town, South Africa; Division of Clinical Pharmacology, Department of Medicine, University of Cape Town, Cape Town, South Africa; Desmond Tutu TB Centre, Department of Paediatrics and Child Health, Faculty of Medicine and Health Sciences, Stellenbosch University, Cape Town, South Africa; Desmond Tutu TB Centre, Department of Paediatrics and Child Health, Faculty of Medicine and Health Sciences, Stellenbosch University, Cape Town, South Africa; University of Zambia, School of Medicine and Children’s Hospital, University Teaching Hospitals, Lusaka, Zambia; Malawi-Liverpool-Wellcome Research Unit, Blantyre, Malawi; Department of Paediatrics and Child Health, Red Cross War Memorial Children’s Hospital, Cape Town, South Africa; SA-MRC Unit on Child & Adolescent Health, University of Cape Town, Cape Town, South Africa; Department of Paediatrics and Child Health and Family Centre for Research with Ubuntu (FAM-CRU), Stellenbosch University, Cape Town, South Africa; Tygerberg Children’s Hospital, Cape Town, South Africa; Drugs for Neglected Diseases Initiative, Geneva, Switzerland; Drugs for Neglected Diseases Initiative, Geneva, Switzerland; Division of Clinical Pharmacology, Department of Medicine, University of Cape Town, Cape Town, South Africa; Department of Paediatrics and Child Health and Family Centre for Research with Ubuntu (FAM-CRU), Stellenbosch University, Cape Town, South Africa; Tygerberg Children’s Hospital, Cape Town, South Africa; Division of Clinical Pharmacology, Department of Medicine, University of Cape Town, Cape Town, South Africa

## Abstract

**Objectives:**

Ethambutol protects against the development of resistance to co-administered drugs in the intensive phase of first-line anti-TB treatment in children. It is especially relevant in settings with a high prevalence of HIV or isoniazid resistance. We describe the population pharmacokinetics of ethambutol in children with TB to guide dosing in this population.

**Methods:**

We pooled data from 188 intensively sampled children from the DATiC, DND*i* and SHINE studies, who received 15–25 mg/kg ethambutol daily according to WHO guidelines. The median (range) age and weight of the cohort were 1.9 (0.3–12.6) years and 9.6 (3.9–34.5) kg, respectively. Children with HIV (HIV+; *n *= 103) received ART (lopinavir/ritonavir in 92%).

**Results:**

Ethambutol pharmacokinetics were best described by a two-compartment model with first-order elimination and absorption transit compartments. Clearance was estimated to reach 50% of its mature value by 2 months after birth and 99% by 3 years. Typical steady-state apparent clearance in a 10 kg child was 15.9 L/h. In HIV+ children on lopinavir/ritonavir, bioavailability was reduced by 32% [median (IQR) steady-state *C*_max_ = 0.882 (0.669–1.28) versus 1.66 (1.21–2.15) mg/L). In young children, bioavailability correlated with age. At birth, bioavailability was 73.1% of that in children 3.16 years or older.

**Conclusions:**

To obtain exposure within the 2–6 mg/L recommended range for *C*_max_, the current doses must be doubled (or tripled with HIV+ children on lopinavir/ritonavir) for paediatric patients. This raises concerns regarding the potential for ocular toxicity, which would require evaluation.

## Introduction

Ethambutol is included in the intensive phase of first-line treatment of TB in areas of high HIV prevalence or isoniazid resistance.^1,2^ It inhibits bacterial growth by preventing mycobacterial cell wall synthesis, but the primary reason for inclusion in first-line anti-TB therapy is to protect against resistance to co-administered first-line anti-TB drugs and to treat extensive disease.^1^ Despite the extensive use of ethambutol in children, knowledge of its pharmacokinetics is limited, and more information is required to characterize the impact of factors such as age, weight, HIV infection and drug–drug interactions to better guide management.

Ethambutol is indicated for the paediatric population at 15–25 mg/kg once daily.^3^ Its oral bioavailability is approximately 80%, with plasma protein binding around 70%–80%.^4^ Ingesting food at the same time decreases the rate of ethambutol absorption but not its overall bioavailability.^1,3^ Ethambutol concentrations peak 2–4 h after a dose. A *C*_max_ of 2–6 mg/L for a mean ethambutol dose of 25 mg/kg has been proposed as a therapeutic target in adults.^3,5^ Following oral administration in healthy subjects, 50%–70% of the dose is excreted in the urine unchanged. The alcohol dehydrogenase enzyme is responsible for the breakdown of ethambutol to an aldehyde intermediate and dicarboxylic acid.^6,7^

HIV and TB co-infections are very common in sub-Saharan Africa.^8^ Children living with HIV (HIV+) receive ART and often require co-treatment for TB, increasing the chance of drug–drug interactions. The commonly reported drug–drug interactions are between rifampicin and either PIs or NNRTIs.^9^ Limited data are available about drug–drug interactions between ethambutol and ART, particularly in children. Another consequence of TB and HIV co-infections is drug–disease interactions. One aspect of drug–disease interactions is malabsorption due to intestinal malfunction. Decreased absorption of antibacterial agents is linked to progressive immunodeficiency.^10^

Pharmacokinetic studies are often small with limited power to detect important covariates. Pooling of data from different studies using non-linear mixed-effects modelling potentially increases the power to detect covariate effects. The aim of the present study was therefore to pool pharmacokinetic data to characterize the population pharmacokinetics of ethambutol in African children, both with and without HIV, treated for TB, thereby identifying factors impacting ethambutol pharmacokinetics in order to guide dosing.

## Methods

### Clinical studies and data

This analysis was performed with plasma concentration data obtained from 188 intensively sampled children from three clinical studies: (i) the pharmacokinetics of lopinavir when super-boosting with ritonavir in infants and young children co-infected with HIV and TB, sponsored by the Drugs for Neglected Diseases *initiative* (DND*i*), which was designed to test whether boosting with additional ritonavir added to co-formulated lopinavir/ritonavir (4:1) to achieve a 4:4 ratio would overcome the rifampicin effect on lopinavir exposures; pharmacokinetic samples were drawn at steady state, before an observed dose and after 1, 2, 4, 6 and 10 h;^11^ (ii) Optimal Dosing of 1st Line Antituberculosis and Antiretroviral Drugs in Children (DATiC),^3,12^ which aimed to assess the pharmacokinetics of first-line anti-TB drugs, and 8 hourly dosing of lopinavir/ritonavir co-administered with rifampicin-based anti-TB treatment; pharmacokinetic samples were collected at steady state pre-dose and at 1, 2, 4, 6 and 8 h post-dose; and (iii) shorter treatment for minimal TB in children (SHINE), which evaluated a 4 month regimen versus the standard 6 month regimen in children dosed with new fixed drug combination (FDC) tablets according to WHO guidelines. Pharmacokinetic samples were collected before drug intake and then at 1, 2, 4, 6, 8 and 12 h after drug intake. All three studies had South African sites, while DATiC included children in Malawi, and SHINE included children in Zambia.^13^ In all the studies, ethambutol was administered to children in the morning in doses of 15–25 mg/kg/day in line with WHO guidelines. The children fasted for at least 1 h prior to receiving a dose of ethambutol. Children from the DND*i* study were dosed using 400 mg tablets (Sandoz Pharmaceuticals Ltd), while those in SHINE received 100 mg tablets (Macleods Pharmaceuticals Ltd) or, for children weighing ≥25 kg, FDC tablets were used, with 275 mg ethambutol per tablet, plus rifampicin, isoniazid and pyrazinamide (Macleods Pharmaceuticals Ltd). In DATiC, children received 100 mg ethambutol tablets (89% had tablets made by RIEMSER Pharma GmbH, and tablets by Sandoz Pharmaceuticals Ltd were used in the remainder). Younger children received crushed tablets due to inability to ingest the full tablet. Ninety-three percent of the latter were below 5 years of age, and all children (*n *= 15) administered ethambutol through a nasogastric tube (NGT) were below 1 year of age. Biochemistry was not routinely monitored in children on the first-line regimen and measures of renal function were not available for this analysis.

### Analyses

Blood samples were collected in EDTA-coated tubes and centrifuged to separate the plasma before freezing at −80°C within 30 min of sampling. Ethambutol concentrations were determined at the Clinical Pharmacokinetic Laboratory at the University of Cape Town. The LC-MS assay was validated over the concentration range of the lower limit of quantification (LLOQ) of 0.0844 mg/L to the upper limit of quantification (ULOQ) of 5.40 mg/L.^14^

### Population pharmacokinetic analysis

Non-linear mixed-effects modelling implemented in NONMEM version 7.4.4 (ICON Development Solutions, Ellicott City, MD, USA) was used to describe ethambutol plasma concentration data. First-order conditional estimation with eta–epsilon interaction (FOCE-I) was employed to estimate population pharmacokinetic parameters of ethambutol. Data visualization and evaluation of NONMEM output were conducted with PsN, Pirana and R Package xpose4.^15^

Ethambutol was administered as ethambutol dihydrochloride (molecular weight = 277.23 g/mol)^16^ and the dose was expressed as mg of this salt in all studies. To obtain the equivalent dose of ethambutol (molecular weight = 204.31 g/mol),^16^ the active compound measured in the assays, the dose of ethambutol dihydrochloride was multiplied by 0.737.

Data from each study were explored separately and included in the model consecutively based on the most intensive data, as suggested by Svensson *et al*.^17^ With the inclusion of each dataset, the model fit was reassessed, and adjusted based on the general principles of model development outlined below.

Single- and multi-compartment models with first-order absorption and elimination were evaluated to identify the model that best described ethambutol concentration data. Lag time and transit compartments^18^ were evaluated for the modelling of absorption. Between-subject variability (BSV) and between-occasion variability (BOV) of random effects were assumed to have log-normal distributions and were modelled using an exponential error term.^19^ The estimates of BSV and BOV were provided as percentage coefficient of variation (%CV). A combined additive and proportional error model was used to describe residual unexplained variability, with the additive error for all samples set to at least 20% of the LLOQ. In DND*i* and SHINE, values were received as censored at the LLOQ, whereas in DATiC, values were received as censored if undetectable and the limit of detection was set to 30% of LLOQ.^20^ To account for the larger level of uncertainty in the imputed censored values, their additive error was inflated by half the censoring threshold.

Censored plasma concentration values were handled with the M6 method,^21^ whereby the last censored value in a series during the absorption phase and the first censored value in a series in the terminal phase were imputed to half of the censoring threshold, while the other censored values in a series were excluded from the model fit and only included in diagnostic plots. The impact of the method chosen to handle values below the limit of quantification (BLQ) on the parameter estimates was tested by repeating the analysis after excluding these points, and no significant change was observed. Model building was guided by the drop in the objective function value (ΔOFV; proportional to −2 log-likelihood), inspection of goodness-of-fit plots, visual predictive check (VPC), biological plausibility and clinical relevance. A decrease in OFV of more than 3.84 between two nested models after the addition of one parameter was considered significant (corresponding to *P *< 0.05).

### Investigating factors that influence ethambutol pharmacokinetics

The effect of body size on pharmacokinetics of ethambutol was introduced by allometrically scaling all clearance and volume parameters. Exponents were fixed to ¾ for clearance and 1 for volume of distribution parameters.^22–24^ Fat-free mass (FFM) and total body weight (TBW)^25^ were assessed as possible size descriptors on disposition parameters. After allometric scaling was included, the enzyme maturation process and its effect on clearance was evaluated according to the approach previously described by Anderson and Holford.^24^ Maturation was calculated as:

(1) $$maturation=\frac{PMAGE^{\gamma }}{\left(PMAGE_{50}^{\gamma }+PMAGE^{\gamma }\right)}$$

where PMAGE denotes postmenstrual age, PMAGE_50_ is PMAGE at which 50% of the maturation is complete, and γ is a parameter determining the shape of the relationship. Gestational age was used to calculate PMAGE; when unavailable, a gestational age of 39 weeks was used. To stabilize the model, we included informative priors^26^ based on a published human renal function maturation model^27^ with 10% uncertainty. Additionally, the effect of age on absorption parameters was tested using different relationships, such as linear, exponential and ‘hockey-stick’ models. After inclusion of weight and age in the model, additional covariates were screened based on the range of covariate values in the dataset, physiological plausibility and inspection of parameter-versus-covariate plots and retained based on statistical significance at *P < *0.01.

Different methods of drug administration were used in the studies due to the inability of younger children to ingest solid dosage forms; age was then tested on absorption parameters. For categorical covariates identified, the fractional change in the typical parameter value was determined. The precision of the final parameter estimates was then evaluated by the sampling importance resampling (SIR) method.^28^

### Simulations

Using Monte Carlo simulations, the final model was used to simulate the AUC_0–24_ and *C*_max_ achieved by each weight band under different dose administrations, including the currently recommended 15–25 mg/kg dose, and these were compared with the proposed therapeutic *C*_max_ of 2–6 mg/L and AUC_0–24_ of 16–29 mg · h/L taken from previously published ethambutol studies in adults.^29–31^ The exposures were simulated using an *in silico* population (*n* > 50 000) weighing 3–35.9 kg with combinations of weight and age previously reported in TB-infected children.^32^ The children were dosed every 24 h.

## Results

The available data consisted of 1012 ethambutol plasma concentrations from 188 children receiving a median (range) dose of 20.2 (12.5–25.0) mg/kg. The median (range) age and weight were 1.9 (0.3–12.6) years and 9.6 (3.9–34.5) kg, respectively. A total of 121 (12%) plasma concentrations (mostly trough samples) were BLQ. Of 188 children in the analysis, 103 (54.8%) were HIV+ on ART. Ninety-two percent of the children on ART were on lopinavir/ritonavir, with 88% on super-boosted lopinavir and 12% on adjusted doses of lopinavir/ritonavir three times daily.^32^ The detailed patient demographics of each study are provided in Table 1.

**Table 1.** Clinical characteristics of patients and demographics in studies included in the analysis

Characteristic	DATiC	DND*i*	SHINE	Combined
All patients/male patients, *n*	79/33	84/36	25/16	188/85
PK samples, *n*	368	471	173	1012
Age (years), median (range)	2.6 (0.3–11.6)	1.6 (0.3–6.8)	3.1 (0.3–12.6)	1.9 (0.3–12.6)
Weight (kg), median (range)	11.1 (4.2–26.7)	8.8 (3.9–14.9)	11.8 (6.4–34.5)	9.6 (3.9–34.5)
FFM (kg), median (range)	9.1 (3.5–22.6)	6.8 (3.3–13.6)	9.2 (4.6–29.2)	7.7 (3.3–29.2)
Ethambutol formulation, *n*^a^				
RIEMSER Pharma GmbH^®^	70	0	0	70
Sandoz^®^	9	84	0	93
Macleods^®^	0	0	25	25
Form of administration				
Whole tablet	27	Not recorded^b^	12	41
Crushed and swallowed	23	Not recorded^b^	13	117
Crushed and syringe	14	0	0	14
Crushed and NGT	15	0	0	15
HIV+, *n*	19	84	0	103
Absolute CD4 count (cells/mm^3^), median (IQR)	576 (214–1318)	893 (463–1730)	0	848 (418–1716)
Viral load (log_10_ copies/mL), median (IQR)	4.36 (3.11–5.72)	5.68 (4.65–6.24)	0	5.51 (4.20–6.21)
Concomitant antiretrovirals				
LPV/r* *+* *ABC* *+* *3TC	11	84	0	95
NVP* *+* *ZDV* *+* *3TC	3	0	0	3
EFV* *+* *ZDV* *+* *3TC	3	0	0	3
EFV* *+* *ABC* *+* *3TC	2	0	0	2

3TC, lamivudine; ABC, abacavir; ZDV, zidovudine; EFV, efavirenz; LPV/r, lopinavir/ritonavir; NVP, nevirapine.

a Number of children on the formulation on the day of the PK visits.

b Whole tablet was imputed for children older than 5 years, while crushed tablet was imputed for children younger than 5 years.

### Structural model and parameter estimates

**Figure 1.**
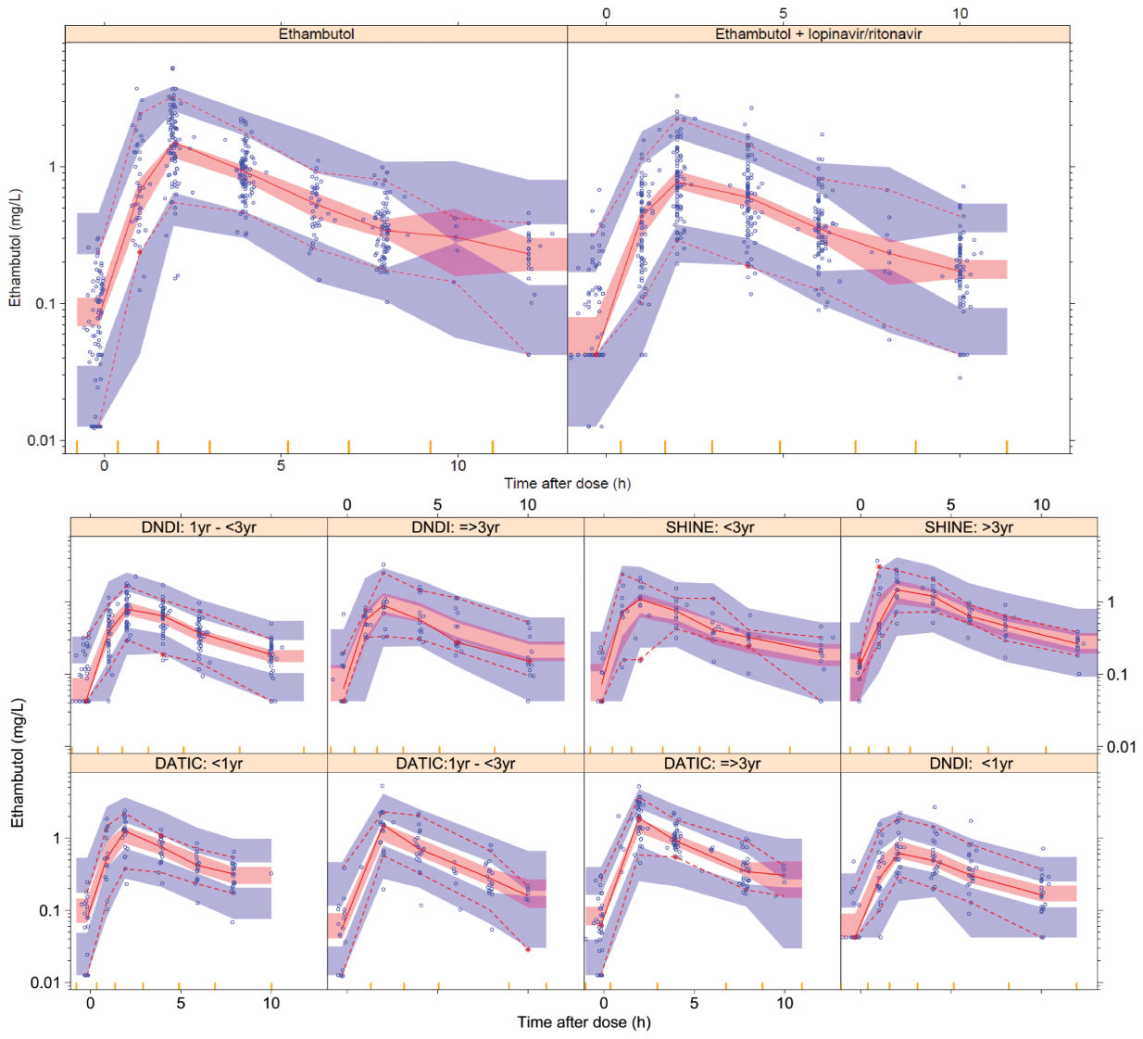
VPC of ethambutol concentration versus time after dose, stratified by HIV treatment (top) and age (bottom). The solid and dashed lines represent the 5th, 50th and 95th percentiles of the observed data, while the shaded areas represent the model-predicted 95% CIs for the same percentiles. The dots are the observed concentrations. This figure appears in colour in the online version of *JAC* and in black and white in the print version of *JAC*.

A two-compartment disposition model with first-order elimination proved superior to a one-compartment disposition model with first-order elimination (ΔOFV = −110; *P *< 10^−6^). The absorption phase was described successfully by a transit compartment model with five transit compartments (ΔOFV = −43.5; *P *< 10^−6^ when compared with simple first-order absorption). The final parameter estimates with uncertainty are presented in Table 2 and a VPC showing suitable fit is shown in Figure 1. The NONMEM control stream is available as Supplementary data at *JAC* Online.

**Table 2.** Parameter estimates of the final model of ethambutol

Model parameter	Typical value		Variability	
	Value	95% CI^a^	% CV	95% CI
CL (L/h)^b^	15.9	14.8–17.3	11.6 (BSV)	7.12–15.3
Central *V*_d_ (L)^b^	44.3	37.2–51.1		
Relative oral bioavailability (F)	1	Fixed	33.1 (BOV)	30.1–36.1
First-order absorption rate constant (ka, h^−1^)	1.43	1.10–1.84	63.7 (BOV)	51.3–77.5
Mean absorption transit time (MTT, min)	40.4	35.3–46.1	48.5 (BOV)	41.2–56.9
Number of absorption transit compartments (*n*)	4.82	3.70–6.32		
Inter-compartmental clearance (L/h)^b^	11.5	10.1–13.2		
Peripheral *V*_d_ (L)^b^	86.2	73.6–102		
PMAGE_50_ (months)	10.8	9.66–11.7		
γ-shape of maturation function	3.25	2.76–3.77		
Change in speed of absorption in DND*i* and SHINE (%)^c^	−23.6	−31.6 to −14.4		
Change in F when on LPV/r (%)	−32.0	−38.9 to −23.8		
Breakpoint for age effect on F (years)	3.16	2.18–4.14		
Age on F, fractional change (year)	+0.0853	+0.0463 to +0.130		
Scaling of BOV in F for unobserved doses (fold change)	1.37	1.12–1.65		
Additive error (μg/L)^d^	16.8	Fixed		
Proportional error (%)	17.7	16.5–19.1		

Of note, to compare the value of all disposition parameters from this analysis with previous reports that may not have accounted for dose conversion from ethambutol dihydrochloride to ethambutol, it is necessary to multiply by 1.357.

a The 95% CI of parameter estimates was obtained with SIR (*n *=* *1000) of the final model.

b All clearances (CL) and volumes of distribution (*V*_d_) were allometrically scaled and the typical values reported here refer to a child weighing 10 kg (at fully mature clearance) not co-treated with lopinavir/ritonavir.

c *Ka* = *θ*_*Ka*_ × *θ*_*Change in speed of absorption*_; $\;MTT=\theta_{MTT}÷\theta_{Change\;in\;speed\;of\;absorption}$.

d Additive error was fixed to 20% of LLOQ; the additive error was not statistically significant from the lower bound (20% of LLOQ) and was fixed to that value.

Inclusion of allometric scaling improved the model fit and FFM was a better size descriptor then TBW (ΔOFV of 12.1 for FFM as opposed to only 6.91 for TBW). The effect of age on clearance was captured using a maturation function. Its inclusion improved the model fit (ΔOFV = −22.5; *P *< 10^−5^), though it was unstable; estimates were unreasonably small. Clearance was predicted to reach 50% of its mature value at around 2 months after birth and 99% of full maturation by around 3 years. The estimated typical ethambutol apparent clearance at steady state, assuming full maturation, was 15.9 L/h in a child weighing 10 kg not co-treated with lopinavir/ritonavir. Of note, to compare the value of the apparent clearance (and other disposition parameters) from this analysis with previous reports that may not have accounted for dose conversion from ethambutol dihydrochloride to ethambutol, it is necessary to multiply by 1.357.

Amongst the examined covariates after the inclusion of allometric scaling and maturation, children on lopinavir/ritonavir had 32% lower ethambutol bioavailability and thus lower overall exposure (ΔOFV = −31.4; *P *< 10^−6^). Children from the DND*i* and SHINE studies exhibited 23.6% slower ethambutol absorption in comparison with the DATiC study (ΔOFV = −13.4; *P *< 10^−4^). The effect of age on absorption parameters was tested using a hockey-stick model, in which the relationship was linear from birth up to a certain cut-off (breakpoint) age, beyond which the absorption parameter value remained constant. Using this approach, the model predicted bioavailability to decrease by 8.5% per year below 3.2 years (breakpoint) (ΔOFV = −9.3; *P *< 10^−3^). The estimate of the additive error hit the stipulated lower boundary (20% of LLOQ), so it was fixed to this value. A 1.37-fold larger BOV in bioavailability in the pre-dose concentrations (ΔOFV = −7.7; *P *< 10^−3^) was observed.

### Monte Carlo simulations

**Figure 2.**
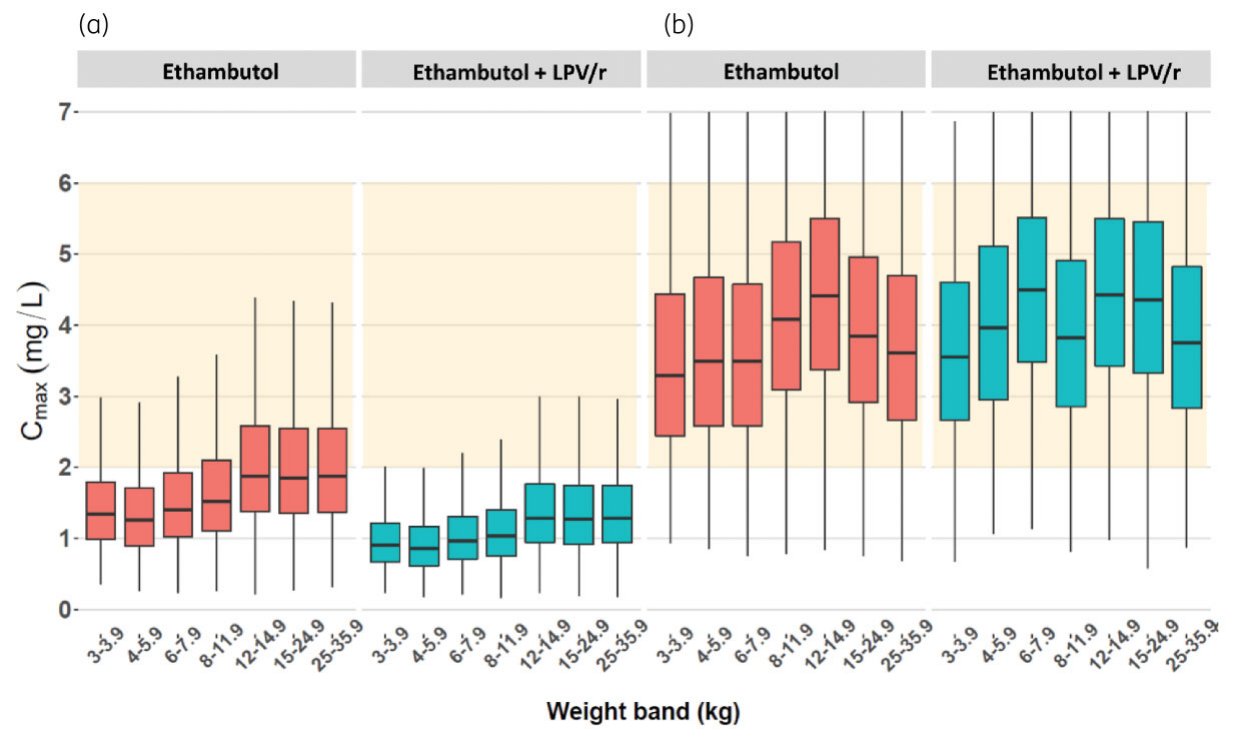
Simulated ethambutol *C*_max_ versus body weight, with the concentrations achieved with the WHO-recommended weight-based dosing (a) and the suggested optimized dosing (b). The red boxes represent patients on ethambutol without co-administration of lopinavir/ritonavir while the blue boxes represent patients to whom ethambutol was co-administered with lopinavir/ritonavir. The orange shaded area from 2 to 6 mg/L represents the recommended range. The box indicates the IQR, while the whiskers denote the 2.5th and the 97.5th percentiles. This figure appears in colour in the online version of *JAC* and in black and white in the print version of *JAC*.

**Figure 3.**
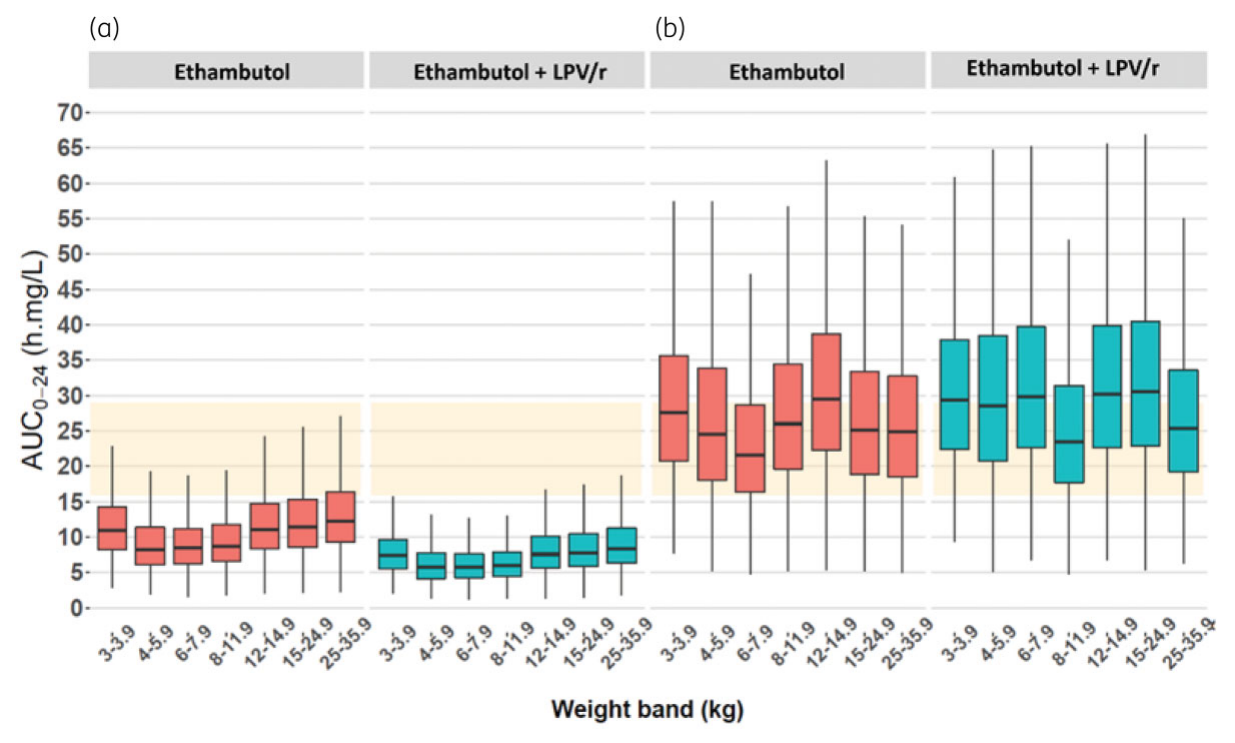
Simulated ethambutol AUC from time 0 to 24 h (AUC_0–24_) versus body weight, with the concentrations achieved with the WHO-recommended weight-based dosing (a) and the suggested optimized dosing (b). The red boxes represent patients on ethambutol without co-administration of lopinavir/ritonavir while the blue boxes represent patients to whom ethambutol was co-administered with lopinavir/ritonavir. The orange shaded area from 16 to 29 mg·h/L represents the reported adult median AUC_0–24_. The box indicates the IQR, while the whiskers denote the 2.5th and the 97.5th percentiles. This figure appears in colour in the online version of *JAC* and in black and white in the print version of *JAC*.

Model-based simulations were used to explore the exposure obtained with the WHO-recommended weight-based dosing. As seen in Figures 2 and 3, most children treated with these guidelines are not predicted to achieve the range of AUC_0–24_ and *C*_max_ observed in adults. To obtain those target exposures, the model predicts that children not on lopinavir/ritonavir will require an average of 50 mg/kg/day, whereas children co-administered lopinavir/ritonavir will require an average of 84 mg/kg/day. Overall, children in the lower weight bands receiving ethambutol will require higher doses compared with children in the upper weight bands. Further details are given in Figures 2 and 3, as well as in Table 3.

**Table 3.** Doses used in simulation of ethambutol hydrochloride exposures

Weight band (kg)	WHO-recommended dosing in mg (mg/kg)^a^	Ethambutol optimised dosing in mg (mg/kg)^b^	
	All children	No LPV/r-based ART	LPV/r-based ART
3–3.9	75 (19–25)	200 (51–67)	300 (77–100)
4–5.9	100 (17–25)	300 (51–75)	500 (85–125)
6–7.9	150 (19–25)	400 (50–66)	800 (100–133)
8–11.9	200 (17–25)	600 (50–75)	800 (66–99)
12–14.9	300 (20–25)	800 (53–67)	1200 (80–100)
15–19.9	400 (20–26)	800 (40–53)	1600 (80–106)
20–24.9	400 (16–20)	800 (32–40)	1600 (64–80)
25–35.9	600 (20–24)	1200 (40–48)	1600 (43–64)

a Current dosing recommendation by WHO.

b Model-based optimized dosing recommendation.

## Discussion

**Figure 4.**
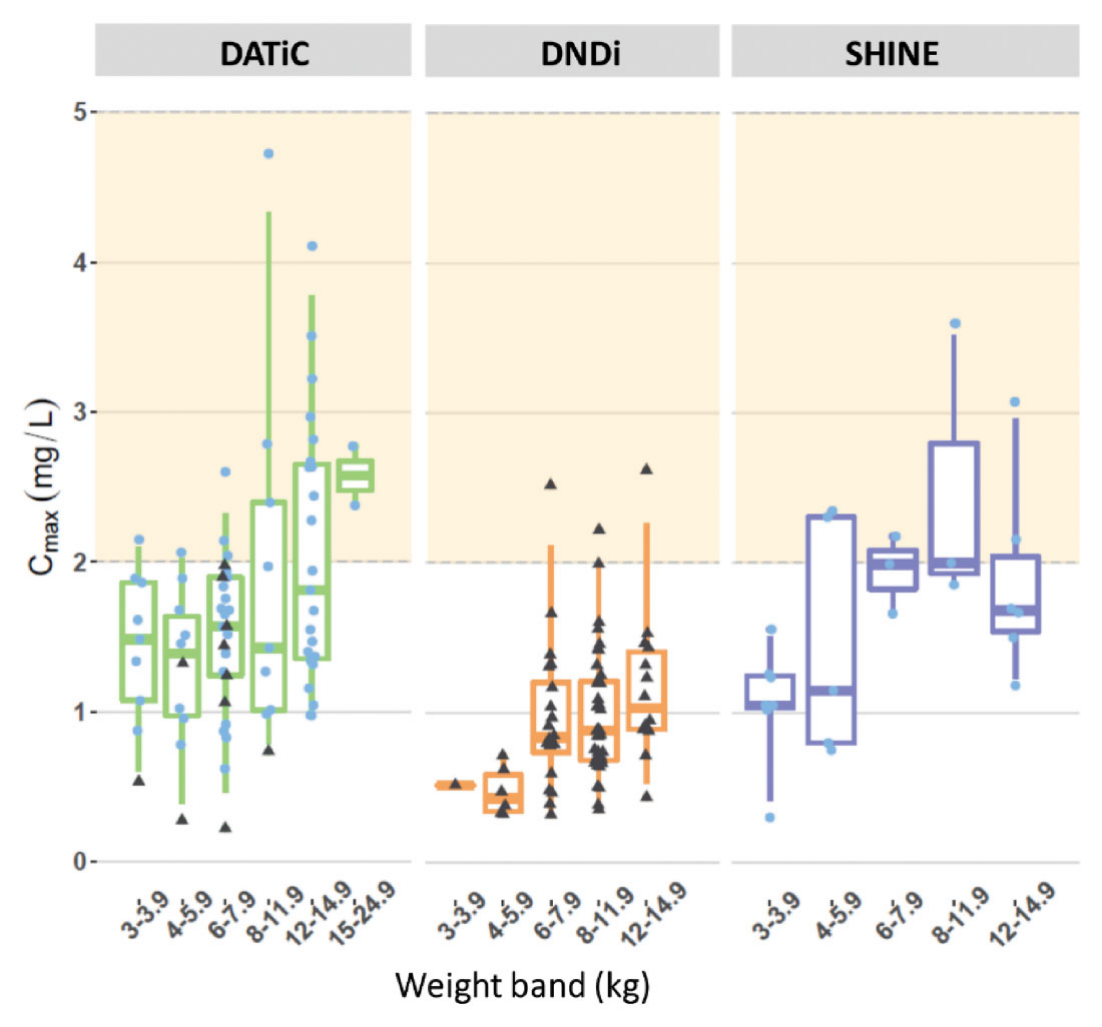
Summary of model-predicted ethambutol *C*_max_ versus body weight for DATiC (left), DND*i* (middle) and SHINE (right). The orange shaded area from 2 to 6 mg/L represents the recommended thresholds. Each dot represents an individual *C*_max_, the black triangular dots represent patients who also received lopinavir/ritonavir and the blue dots represent co-administration without lopinavir/ritonavir. The box indicates the IQR, while the whiskers denote the 2.5th and the 97.5th percentiles. This figure appears in colour in the online version of *JAC* and in black and white in the print version of *JAC*.

**Figure 5.**
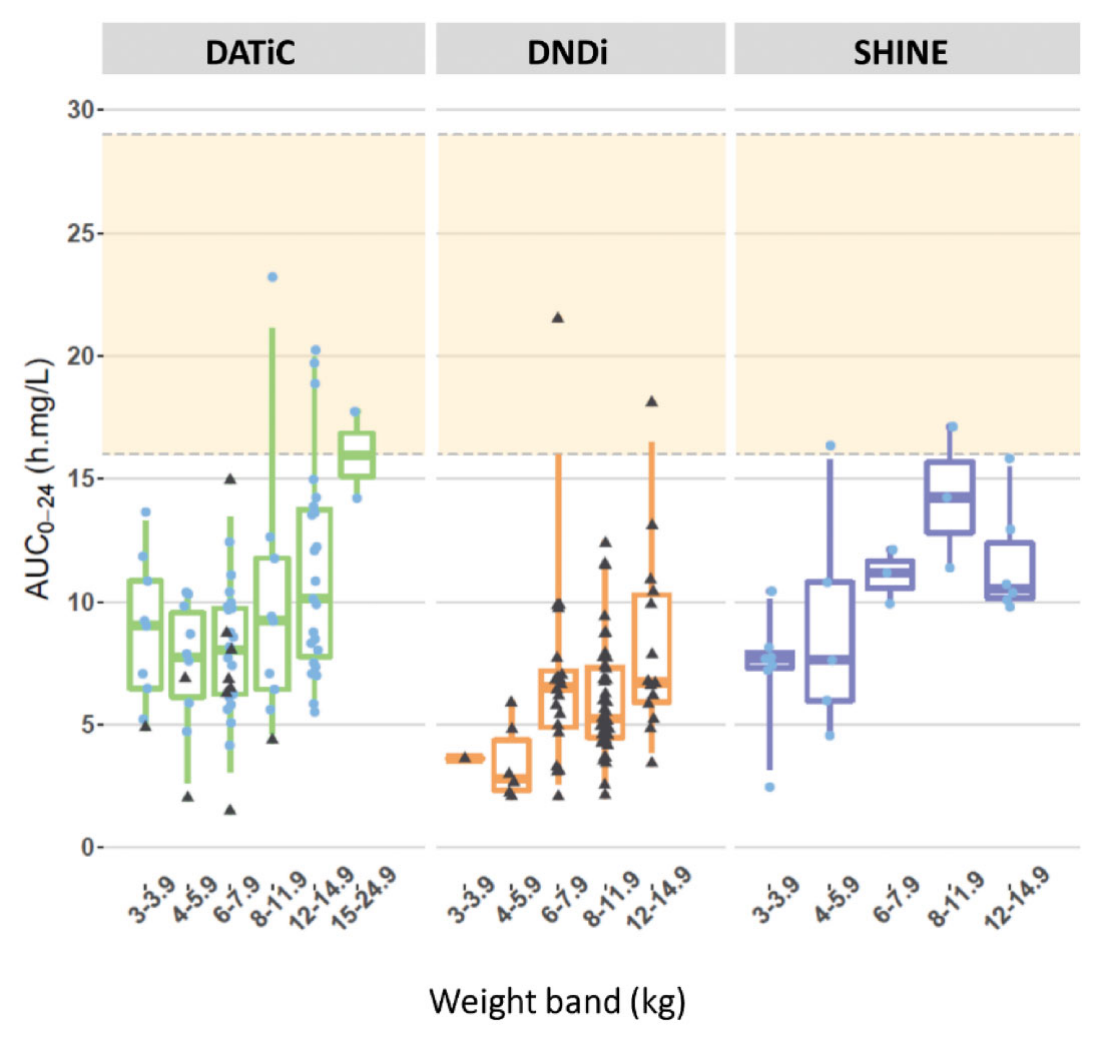
Summary of model-predicted ethambutol AUC from time 0 to 24 h (AUC_0–24_) versus weight for DATiC (left), DND*i* (middle) and SHINE (right). The orange shaded area from 16 to 29 mg · h/L represents the range of study medians reported for adults. Each dot represents an individual AUC, the black triangular dots represent patients who also received lopinavir/ritonavir and the blue dots represent co-administration without lopinavir/ritonavir. The box indicates the IQR, while the whiskers denote the 2.5th and the 97.5th percentiles. This figure appears in colour in the online version of *JAC* and in black and white in the print version of *JAC*.

In this population pharmacokinetic meta-analysis of ethambutol in children, the effects of weight, age and HIV infection were explored. The observed ethambutol concentrations were lower than the suggested adult target concentrations, i.e. 2 h *C*_max_ of 2–6 mg/L; see Figures 4 and 5.^3^ In children receiving a median ethambutol dihydrochloride dose of 20.2 mg/kg, the model-predicted median (IQR) steady-state ethambutol *C*_max_ was 1.66 (1.21–2.15) mg/L at 2.74 h for children not taking lopinavir/ritonavir, while the value was 0.882 (0.669–1.28) mg/L at 3.0 h for children co-treated with lopinavir/ritonavir. The predicted ethambutol concentrations were comparable with those from other paediatric studies, which are also low, ranging from 0.78 to 2.1 mg/L when dosing at 10 to 20 mg/kg.^3,33–35^ It is worth noting that direct comparison of *C*_max_ values between these pharmacokinetic studies is challenging, since different types of assays for measurement of drug concentration, sampling schedules and types of formulations were used. These variations can heavily influence *C*_max_ and *T*_max_, and may account for the apparently faster rate of absorption (resulting in shorter *T*_max_ and higher *C*_max_) that we found in DATiC compared with the other studies.

The model identified a 32% reduction in ethambutol exposure in HIV+ children on lopinavir/ritonavir. As most children on ART received lopinavir/ritonavir, we could not robustly compare the effects of different antiretrovirals. Although the currently available studies are conflicting in their results, HIV+ patients have been shown to achieve somewhat lower concentrations of ethambutol.^3,31,34,36^ In non-compartmental analyses of ethambutol pharmacokinetics in children, the HIV+ groups had lower AUC than HIV-negative groups.^3,36,37^ Two of these studies also reported significant reduction of *C*_max_ in the HIV+ group compared with the HIV-negative group.^3,36^ The children in these studies were dosed according to the WHO 2010 guidelines and included children on ART without lopinavir/ritonavir. The exact mechanism causing the low ethambutol concentrations in HIV+ patients is unclear. While anti-TB drugs, particularly rifampicin, through activation of the pregnane X-receptor, cause significant interactions with PIs and NNRTIs, there is currently limited evidence describing potential drug–drug interactions between ethambutol and ART. Ethambutol is a substrate of the P-glycoprotein efflux pump^38^ and ritonavir induces P-glycoprotein through activation of the pregnane X-receptor, increasing the amount transported back into the gut lumen, reducing the bioavailability.^39–41^ Other potential causes of low drug concentrations in advanced HIV infection are malabsorption and low serum albumin.^42–44^ The association of HIV with reduced concentrations has been noted to be more profound for ethambutol and rifampicin than other first-line drugs.^45^ In our analysis, the majority of children on lopinavir/ritonavir were administered ethambutol formulations different to our reference child, so it is also possible that the reduced exposure observed in children on lopinavir/ritonavir is due to the type of formulation this group used. However, when testing this effect of lopinavir/ritonavir on ethambutol exposure only within the DATiC study, where all children received the same formulation, the reduced ethambutol exposure was still observed. Whether the decreased ethambutol exposures seen in children with super-boosted lopinavir are indeed associated with HIV-related malabsorption, drug–drug interaction or formulation (or study) differences could not be established with certainty in this analysis; the exact mechanism driving this effect and its clinical relevance needs further investigation.

The decreased bioavailability observed in children below 3 years of age could be related to the drug administration methods used in these age groups. While older children can swallow tablets or capsules, this is not always the case for the younger children, who require tablets to be split or crushed. The problem with this method is ensuring the correct dose is measured and given to the child by the caregiver; such methods increase the variability in the dose administered.^46^ One limitation in our analysis is the lack of information on the method of administration in some children. For the DND*i* study, the method of administration was imputed based on age. Both age and method of administration were tested in the model to explain the observed lower ethambutol bioavailability. Age performed better and was therefore retained in the final model.

The simulations predict that paediatric patients treated according to the currently recommended 15–25 mg/kg/day will achieve concentrations lower than the target AUC and *C*_max_ values. For the majority of children to obtain exposure within the target range, the doses require doubling if not on lopinavir/ritonavir, and tripling if on lopinavir/ritonavir. However, such adjustments to the doses of ethambutol raise the concern of dose-dependent ocular toxicity. Although uncommon, ocular toxicity has been reported in adults on daily ethambutol doses of 15–25 mg/kg, with more common occurrences at doses greater than 50 mg/kg. In children, ocular toxicity is rare at daily ethambutol doses of 15–30 mg/kg.^47^ This is possibly due to the low ethambutol exposures in children compared with adults. Further studies are needed to substantiate whether the currently recommended doses in children offer sufficient exposure to protect companion drugs against resistance or whether increased ethambutol doses would be required.

Our analysis has several limitations. Incomplete information on creatinine values, which were not collected for all children, limited our ability to assess whether renal function could explain some of the variability in clearance. HIV+ patients were on ART so we could not distinguish whether the observed decreased bioavailability was due to treatment or disease.

To conclude, ethambutol doses of 15–25 mg/kg/day, as advised in the current WHO guidelines, resulted in *C*_max_ and AUC values lower than those reported in adults. Moreover, exposures were 32% lower in children co-treated with lopinavir/ritonavir. Decreased bioavailability was also seen in children below 3 years of age. Considerably higher ethambutol doses are needed in children to match adult exposures, but concerns about the potential for ocular toxicity limit this approach and, moreover, with a range of drugs for TB now available, a fourth drug with a potentially better risk–benefit profile could be considered. The consequences of failing to achieve sufficiently high ethambutol concentrations in children need further investigation.

## Supplementary Material

dkac127_Supplementary_Data

## Supplementary data

The NONMEM control stream is available as Supplementary data at *JAC* Online.
