# Supplementary material for: Population pharmacokinetics of ethambutol in African children: a pooled analysis
Source: J Antimicrob Chemother. 2022 Apr 25;77(7):1949–59. doi: 10.1093/jac/dkac127 (PMC9633720; doi:10.1093/jac/dkac127)
Supplement: dkac127_Supplementary_Data [file dkac127_supplementary_data.docx]

**Supplementary data**

**Code**

NONMEM control stream:

;; 1. Based on: run500a

;; 2. Description: using gestation for maturation

;; x1. Author: Tjokosela Tikiso

; Settings for the memory of NONMEM

$SIZES      PD=-1000 LVR=-150 *LTH*=-200 MAXFCN=10000000 LNP4=-150000

$PROBLEM    DATIC_ETH

$INPUT      ID OCC TIME VPC_TIME EVID AMT SS II DV MDV BLQ STUDY WT HT *AGE* SEX HAZ WAZ

            BAZ BMI ALB HEMO HIV *ALT* BIOCREAT BIOBILI

             LPVRTV ADMIN SITE_ID EFV NVP ABC X3TC ZDV MULTIVIT

            IRON GESTATION ADJ_ADMIN STUDY_AGE

; IGNORE=@ will skip any line starting with any non-numerical character

$DATA   data.dat IGNORE=@

$SUBROUTINE *ADVAN13* *TRANS1* TOL=9 ; TOL is the precision to solve differential equations

; ATOL=6 ; absolute tolerance, 10^ATOL of your dose unit. A lower value makes the model run faster

; Remember that ATOL is only going to work in $SUBROUTINE from 7.4.1, otherwise include ATOL in $ESTIMATION

; SSTOL=3 SSATOL=3 ; This is the tolerance used when calculating steady-state. Put a low value to avoid the model running forever.

; Options SSTOL and SSATOL are only going to work in 7.4.1 or later

$MODEL

NCOMPARTMENTS = 3 ; 3  ; 4

COMP=(*ABS* DEFDOSE)

COMP=(CENTRAL DEFOBSERVATION)

COMP=(PERI1)

;------------------------------------------------------------------------------------------------------------------------------------------

;PRIOR--------------------------------------------------

;Sim_start

$PRIOR      NWPRI NPEXP=1 *PLEV*=0.9999

;Sim_end

$PK

; ---------- BOV

BOVCL = ETA(9)

BOVBIO = ETA(11)

BOVKA = ETA(13)

BOVMTT = ETA(15)

*IF* (OCC==2) *THEN*

    BOVCL = ETA(10)

    BOVBIO = ETA(12)

    BOVKA = ETA(14)

    BOVMTT = ETA(16)

*ENDIF*

; ------- BSV

BSVCL   = ETA(1)

BSVV   = ETA(2)

BSVKA   = ETA(3)

BSVBIO = ETA(4)

BSVCLMET = ETA(5)

BSVVMET = ETA(6)

BSVQ   = ETA(7)

BSVVP   = ETA(8)

SCALE_BOVBIO = 1

*IF* (OCC==1) SCALE_BOVBIO=THETA(18)

BOVBIO = SCALE_BOVBIO*BOVBIO

; -------------- Calculation of Fat-free Mass

; Conversion from cm to m

HTM = HT/100

; These formulas require WT in KG and HT in m, and AGE in years !!!

; Formulas for Children

*IF* (SEX*.EQ.*0) *THEN*  ; female ; IN DATIC females were "1" and males "0" I changed that and made females "0" just as in DNDI

        ALALPHA=1.11 ; lower bound of sigmoid hyperbolic function

        A50=7.1      ; ffm maturation half-time (years)

        ALGAMMA=1.1  ; Sigmoidicity coefficient

        WHSMAX=37.99

        WHS50=35.98

*ELSE*        ;males

        ALALPHA=0.88

        A50=13.4

        ALGAMMA=12.7

        WHSMAX=42.92

        WHS50=30.93

*ENDIF*

HTM2 = HTM**2    ; IMPORTANT: HEIGHT is used in meters!!!

AGEGAM=AGE**ALGAMMA  ; Compute this once and use many times

A50GAM=A50**ALGAMMA  ; Compute this once and use many times

FFM_CH = ((AGEGAM+ALALPHA*A50GAM)/(AGEGAM+A50GAM))*((WHSMAX*HTM2*WT)/(WHS50*HTM2+WT))

FAT_CH = WT-FFM_CH

PERFAT_CH = FAT_CH/WT

; ------- Typical values of covariates

TVWT = 10

TVFAT = 3 ; from datic only

TVFFM = 7.7

TVPERFAT = 0.2; from datic only

;--------- Allometric scaling and covariates

ALLMCL_WT = (WT/TVWT)**0.75

ALLMV_WT = (WT/TVWT)

ALLMCL_FAT_CH = (FAT_CH/TVFAT)**0.75

ALLMV_FAT_CH = (FAT_CH/TVFAT)

ALLMCL_FFM_CH = (FFM_CH/TVFFM)**0.75

ALLMV_FFM_CH = (FFM_CH/TVFFM)

STUDY_ABS = 1

*IF*(STUDY>1) STUDY_ABS= THETA (13); absorption of other studies relative to DATIC

;LPV/r impact on BIO

LPVR = 0

*IF* (LPVRTV>0) LPVR = 1

LPVR_BIO=1

*IF*(LPVR==1) LPVR_BIO= THETA (14)

; EFFECT OF AGE ON BIO

BRK_AGE = THETA(15)

AGE_BIO=1

*IF*(*AGE<*BRK_AGE) AGE_BIO = (1 + THETA(16)*(AGE - BRK_AGE))

*IF*(*AGE>*BRK_AGE) AGE_BIO = (1 + THETA(17)*(AGE - BRK_AGE))

; Maturation of CL

PMAGE = AGE + GESTATION/52

LOGPMAGE50 = THETA(1)

GAMMA = *EXP*(THETA(2))

MATCL=0

*IF* (PMAGE>0) MATCL=1/(1+*EXP*(-GAMMA*(*LOG*(PMAGE)-LOGPMAGE50)))

;---------Typical values--------------------------------------------------------------------------------------------------------------------------------------------------------

TVCL = THETA(3)*ALLMCL_FFM_CH*MATCL

TVV = THETA(4)*ALLMV_FFM_CH

TVKA = THETA(5)*STUDY_ABS

TVBIO = THETA(6)*AGE_BIO*LPVR_BIO

TVMTT = THETA(9)/STUDY_ABS

TVNN = THETA(12)

TVQ = THETA(10)*ALLMCL_FFM_CH

TVVP = THETA(11)*ALLMV_FFM_CH

;----------------------------------------------------------------------------------------------------------------------------------------------------

;-----------Define parameters------------------------------------------------------------------------------------------------------------------------------------------

CL  = TVCL**EXP*(BSVCL+BOVCL) ; CLEARANCE

V   = TVV**EXP*(BSVV) ; CENTRAL VOL.

KA  = TVKA**EXP*(BSVKA+BOVKA) ; ABS. RATE CONSTANT

BIO = TVBIO**EXP*(BSVBIO+BOVBIO) ; BIOAVAILABILITY

MTT = TVMTT**EXP*(BOVMTT) ; MTT TIME

NN  = TVNN ; Number of transit compartments

Q   = TVQ**EXP*(BSVQ)

VP  = TVVP**EXP*(BSVVP)

K = CL/V

K23 = Q/V

K32 = Q/VP

;---------------------------------------------------------------------------------------------------------------------------------------------------------

F1=0 ; I need to set bioavailability in compartment 1 to 0 for this implementation of the transit compartment absorption

KTR = (NN+1)/MTT

*IF* (NEWIND/=2*.OR.*EVID>=3) *THEN* ; new individual, or reset event

    ; The values read here will be stored in TDOS and PD in this very PK call.

    TNXD=TIME ; Time of the dose

    PNXD=AMT ; Amount. If it's zero, the DE is deactivated.

*ENDIF*

TDOS=TNXD ; This will either save here the temporary values if it's a new individual...

PD=PNXD ; ...or the values which were read one record ahead during the execution of the previous record.

*IF*(AMT>0) *THEN* ; This reads one record ahead and stores the data to be used when running the following record

; IF(AMT.GT.0.AND.ALAG1.EQ.0) THEN ; Use this INSTEAD if there is ALAG, as it will also checks if the ALAG is not 0. Note that you normally do not want to include both ALAG and transit, this is a very exceptional case

    TNXD=TIME

    PNXD=AMT

*ENDIF*

PIZZA = *LOG*(BIO*PD*KTR + 0.00001) - GAMLN(NN+1)  ; without +0.00001, it won't work with ETAs in bioavailability

; Initialisation

A_0(1) = 0.000001

A_0(2) = 0.000001

A_0(3) = 0.000001

$DES

TEMPO = T-TDOS ; this is time after dose for the transit, it should always be >= 0

KTT = 0

DADT(1) = -KA*A(1)

*IF*(PD*.GT.*0*.AND.*TEMPO*.GT.*0) *THEN* ; This happens only if PD>0, so only if a dose has been detected

    KTT = KTR*(TEMPO)

    DADT(1) = *EXP*(PIZZA+NN**LOG*(KTT)-KTT) -KA*A(1)

*ENDIF*

DADT(2) = KA*A(1)-K23*A(2)+K32*A(3)-K*A(2)

DADT(3) = K23*A(2)-K32*A(3)

;-------------------------------------------------------------------------------------------------------------------------------------------------------------------------------

$ERROR

; Assays used in Datic

;       LLOQ    min ADD = LLOQ/5    LOD = 30% LLOQ  Imputed undetectable = LOD/2    extra ADD for imputed = LOD/2

;EMB    0.084   0.0168              0.0252          0.0126                          0.0126

;PZA    0.2     0.04                0.06            0.03                            0.03

;INH    0.0977  0.01954             0.02931         0.014655                        0.014655

;ACINH  0.0488  0.00976             0.01464         0.00732                         0.00732

;RIF    0.117   0.0234              0.0351          0.01755                         0.01755

;DESRIF 0.0391  0.00782             0.01173         0.005865                        0.005865

; DEFINE LLOQ VALUE

; LLOQ could be study-specific, e.g if you have data from different labs in your analysis

; In that case, you can use IFs, or you can define the values as covariates in the dataset

LLOQ = 0.084 ; nominal EMB LLOQ = 0.084

; DEFINE censoring threshold (CENS_THR, generally LOD)

; Generally the same as LLOQ, but not if the LLOQ data was released by the lab.

; If censoring threshold is not explicitly indicated, we can generally assume it to be the limit of detection (LOD).

; The signal-to-noise ratio is generally assumed to be 10 at the LLOQ, and 3 at the LOD

; https://en.wikipedia.org/wiki/Detection_limit;

; Keizer RJ, Jansen RS, Rosing H, Thijssen B, Beijnen JH, Schellens JHM, Huitema ADR.

; Incorporation of concentration data below the limit of quantification in population pharmacokinetic analyses.

; Pharmacol Res Perspect [Internet]. 2015 Mar;3(2):e00131. Available from: http://doi.wiley.com/10.1002/prp2.131

CENS_THR=LLOQ

*IF* (STUDY==1) CENS_THR = 0.0252 ; inferred EMB LOD = 30% of LLOQ = 0.0252

IPRED = A(2)/V

IRES = DV-IPRED

PROP = IPRED*THETA(7)

; ADD is defined as 20% of LLOQ + THETA(.)

; The lower bound of THETA(.) can be zero, if it goes there, we can fix it to zero and ADD will be 20% of LLOQ

; REMEMBER that when you report the value of ADD and its uncertainty, you need to work out numbers, as NONMEM gives uncertainty on THETA, not ADD

; An alternative approach is to set the lower bound of the THETA for the additive error to 20% of the LLOQ.

; In that case, one does not have to worry about adjusting the precision.

; On the other hand, this cannot be done if you have different LLOQs within your analysis (e.g. different labs)

ADD = LLOQ/5 + THETA(8)

; For BLQ==1 (i.e. first CENSORED value in a series, which was imputed to CENS_THR/2), we add extra additive error on the concentrations,

; since the value in DV has been imputed and therefore more uncertain.

*IF* (ICALL==2*.AND.*BLQ==1) *THEN*

    ADD = ADD + CENS_THR/2

*ENDIF*

NO_FIT = 0

; For BLQ==2 (i.e. the trailing CENSORED values in a series that were imputed to CENS_THR/2), we don't want these to influence the fit,

; we only want them for simulation-based diagnostics such as the VPC.

; So we define a separate error structure for these points. It has no proportional component

; (PROP = 0, as we would not want these points to affect our estimate of proportional error)

; and a FIXED and HUGE additive component (ADD = 1000000000, large with respect to the readings of concentration),

; so that the values do not affect the fit. It's also a good idea to repeat the diagnostic plots without the BLQ=2 points

*IF* (ICALL==2*.AND.*BLQ==2) *THEN*

    ADD = 1000000000

    PROP = 0

    NO_FIT = 1

*ENDIF*

; Huge error also for all the BLQ in pre-dose, so that it is virtually removed

;IF (ICALL/=4.AND.BLQ>=1.AND.PRE_DOSE==1) THEN

;   ADD = 1000000000

;   PROP = 0

;   NO_FIT = 1

;ENDIF

W = *SQRT*(ADD**2+PROP**2)

; Protective code if the W is 0, but it should never happen in this case, as ADD cannot be 0

*IF* (W*.LE.*0.000001) W=0.000001

IWRES = IRES/W

Y = IPRED + W*ERR(1)

; To prevent simulation (ICALL==4) of negative values. It set a positive lower bound for Y, so that VPCs in the log-scale can be plotted

*IF* (ICALL==4*.AND.*Y<=CENS_THR) Y = CENS_THR/2

; To calculate time after dose.

*IF*(AMT>0) *THEN*

    TIMEDOSE = TIME

    AMOUNTDOSE = AMT

*ENDIF*

TAD = TIME-TIMEDOSE

VARCL = BSVCL + BOVCL

VARBIO = BSVBIO + BOVBIO

VARKA   = BSVKA + BOVKA

VARAUC = BSVBIO + BOVBIO - BSVCL - BOVCL

AA1=A(1)

AA2=A(2)

AA3=A(3)

NGT = 0

*IF* (STUDY==1 .AND. ADMIN==3) *NGT=*1

AGE_LT1 = 0

*IF* (*AGE<*1) AGE_LT1 = 1

HIV_POS = 0

*IF* (HIV==2) HIV_POS = 1; was 2 the only ones with HIV in datic? ; In the dataset all DNDI should have HIV==2 since they were all possitive

STUDY_ADMIN=0

; DATIC

*IF*(STUDY==1 .AND. ADMIN==0) STUDY_ADMIN= 1; WHOLE

*IF*(STUDY==1 .AND. ADMIN==1) STUDY_ADMIN= 2 ;CRUSHED SWALLOWED

*IF*(STUDY==1 .AND. ADMIN==2) STUDY_ADMIN= 3; CRUSHED SYRINGE

*IF*(STUDY==1 .AND. ADMIN==3) STUDY_ADMIN= 4;; NGT

*IF*(STUDY==1 .AND. ADMIN==4) STUDY_ADMIN= 5;TYGERB

DND_ADMIN=0

*IF*(STUDY==2 .AND. *AGE<*3) DND_ADMIN= 1;CRUSHED

*IF*(STUDY==2 .AND. *AGE>*3) DND_ADMIN= 2;FULL

;DNDI

*IF*(STUDY==2 .AND. DND_ADMIN==1) STUDY_ADMIN= 6;ADMIN==0 = FULL TABLETS

*IF*(STUDY==2 .AND. DND_ADMIN==2) STUDY_ADMIN= 7

;SHINE

*IF*(STUDY==3 .AND. ADMIN==0) STUDY_ADMIN= 8;;

*IF*(STUDY==3 .AND. ADMIN==1) STUDY_ADMIN= 9;WHOLE

SITE_ADMIN=0

; D

*IF*(SITE_ID==1 .AND. ADMIN==2) SITE_ADMIN= 1

*IF*(SITE_ID==1 .AND. ADMIN==3) SITE_ADMIN= 2

;M

*IF*(SITE_ID==2 .AND. ADMIN==0) SITE_ADMIN= 3

*IF*(SITE_ID==2 .AND. ADMIN==1) SITE_ADMIN= 4

*IF*(SITE_ID==2 .AND. ADMIN==2) SITE_ADMIN= 5

;X

*IF*(SITE_ID==3 .AND. ADMIN==0) SITE_ADMIN= 6

*IF*(SITE_ID==3 .AND. ADMIN==1) SITE_ADMIN= 7

*IF*(SITE_ID==3 .AND. ADMIN==2) SITE_ADMIN= 8

;T

*IF*(SITE_ID==4 .AND. ADMIN==4) SITE_ADMIN= 9

;DNDI

*IF*(SITE_ID==5 .AND. ADMIN==6) SITE_ADMIN= 10

SITE_ADMIN_OC2=0

; D

*IF*(OCC==2 .AND. SITE_ID==1 .AND. ADMIN==2) SITE_ADMIN_OC2= 1

*IF*(OCC==2 .AND. SITE_ID==1 .AND. ADMIN==3) SITE_ADMIN_OC2= 2

;M

*IF*(OCC==2 .AND. SITE_ID==2 .AND. ADMIN==0) SITE_ADMIN_OC2= 3

*IF*(OCC==2 .AND. SITE_ID==2 .AND. ADMIN==1) SITE_ADMIN_OC2= 4

*IF*(OCC==2 .AND. SITE_ID==2 .AND. ADMIN==2) SITE_ADMIN_OC2= 5

;X

*IF*(OCC==2 .AND. SITE_ID==3 .AND. ADMIN==0) SITE_ADMIN_OC2= 6

*IF*(OCC==2 .AND. SITE_ID==3 .AND. ADMIN==1) SITE_ADMIN_OC2= 7

*IF*(OCC==2 .AND. SITE_ID==3 .AND. ADMIN==2) SITE_ADMIN_OC2= 8

;T

*IF*(OCC==2 .AND. SITE_ID==4 .AND. ADMIN==4) SITE_ADMIN_OC2= 9

;DNDI

*IF*(OCC==2 .AND. SITE_ID==5 .AND. ADMIN==6) SITE_ADMIN_OC2= 10

;--------------------------------------------------------------------------------------------------------------------------------------------------------------------

$THETA

(-3, -0.102,5) ; 1 PMAGE50 yrs [log]

(-2, 1.15,3) ; 2 GAMMA_AGE [log]

(1, 21.8,60) ; 3 CL [L/h]

(1, 61.3,300) ; 4 V [L]

(0.5, 1.45,5) ; 5 KA [1/h]

(1) FIX ; 6 BIO

(0, 0.177,0.5) ; 7 PROP []

(0, 0,1) FIX ; 8 ADD [mg/L]

(0, 0.681,3) ; 9 MTT [h]

(0.1, 15.8,100) ; 10 Q [L/h]

(0.1, 117,600) ; 11 VP [L]

(-2, 4.86,15) ; 12 NN []

(0.01, 0.782,5) ; 13 DNDI_SHINE_ABS

(0.01, 0.684,5) ; 14  LPVR_BIO

(-4, 3.17,5) ; 15 BRK_AGE

(-1, 0.0963,5) ; 16 SLOPE

(-1, 0,5) FIX ; 17 FLAT_FIXED_LINE

(0.1, 1.37,10) ; 18 PREDOSE BOVBIO []

;**********************DONE

; PRIORS

;Sim_start

$THETAP

-0.08906 FIX; 1 PMAGE50 yrs

 1.23 FIX ; 2 GAMMA_AGE [

;Sim_end

;---------------------------------------

; UNCERTAINTY IN PRIORS

;Sim_start

$THETAPV  BLOCK(2) FIX

0.01  ;

0 0.01

;Sim_end

$OMEGA  BLOCK(1)

 0.0246 ;   1 BSV CL

$OMEGA  BLOCK(1)

 0 FIX  ;    2 BSV V

$OMEGA  BLOCK(1)

 0 FIX  ;   3 BSV KA

$OMEGA  BLOCK(1)

 0 FIX  ;  4 BSV BIO

$OMEGA  BLOCK(1)

 0 FIX  ; 5 BSV CLMET

$OMEGA  BLOCK(1)

 0 FIX  ; 6 BSV VMET

$OMEGA  BLOCK(1)

 0 FIX  ;     7 BSVQ

$OMEGA  BLOCK(1)

 0 FIX  ;    8 BSVVP

;---------------------------------------------------------------------------------------------------------------------------------------------------------------------

$OMEGA  BLOCK(1)

 0 FIX  ;    9 BOVCL

$OMEGA  BLOCK(1) SAME

;----------------------------------------------------------------------------------------------------------------------------------------------------------------------

$OMEGA  BLOCK(1)

 0.199 ;  11 BOVBIO

$OMEGA  BLOCK(1) SAME

;---------------------------------------------------------------------------------------------------------------------------------------------------------------------

$OMEGA  BLOCK(1)

 0.403 ;   13 BOVKA

$OMEGA  BLOCK(1) SAME

;---------------------------------------------------------------------------------------------------------------------------------------------------------------------

$OMEGA  BLOCK(1)

 0.233 ;  15 BOVMTT

$OMEGA  BLOCK(1) SAME

;--------------------------------------------------------------------------------------------------------------------------------------------------------

$SIGMA 1 FIX

;-------------------------------------------------------------------------------------------------------------------------------------------------------

;Sim_start

$ESTIMATION MSFO=run500d.msf MAXEVAL=9999 PRINT=1 METHOD=1 INTER

NOABORT NONINFETA=1 ETASTYPE=1 NSIG=3 ATOL=9 SIGL=9 SADDLE_RESET=1 MCETA=500 RANMETHOD=4P

REPEAT

;$SIMULATION (12345) ONLYSIMULATION
